# Crystal structure and Hirshfeld surface analysis of *N*-{*N*-[amino­(di­methyl­amino)­meth­yl]carbamimido­yl}-3-bromo­benzene­sulfonamide

**DOI:** 10.1107/S2056989023002165

**Published:** 2023-03-21

**Authors:** Kexin Su, Jiangshui Luo, Luc Van Meervelt

**Affiliations:** a Department of Chemistry, KU Leuven, Biomolecular Architecture, Celestijnenlaan 200F, Leuven (Heverlee), B-3001, Belgium; b College of Materials Science and Engineering, Sichuan University, Chengdu, 610065, People’s Republic of China; University of Kentucky, USA

**Keywords:** crystal structure, metformin, hydrogen-bond inter­actions, π–π inter­actions, Hirshfeld surface analysis

## Abstract

The title compound, C_10_H_14_BrN_5_O_2_S, is the bromo­benzene­sulfonamide derivative of the type 2 diabetes drug metformin. The asymmetric unit contains two mol­ecules with almost identical conformations but a different orientation of the bromo­phenyl moiety. Both mol­ecules exhibit intra­molecular N—H⋯N and N—H⋯O hydrogen bonds. The mol­ecular packing features chain formation in the *a*-axis direction by alternating N—H⋯N and N—H⋯O inter­actions. In addition, ring motifs consisting of four mol­ecules and π–π inter­actions between the phenyl rings contribute to the three-dimensional architecture. A Hirshfeld surface analysis shows that the largest contributions to surface contacts arise from contacts in which H atoms are involved.

## Abstract 2

The crystal structure of the bromo­benzene­sulfonamide derivative of the type 2 diabetes drug metformin is presented.

## 1. Chemical context

Metformin is a widely known effective drug for type 2 diabetes, which does not cause weight gain and rarely causes hypoglycemia. Metformin works by decreasing gluconeogenesis in the liver, increasing insulin sensitivity and preventing insulin resistance (Giannarelli *et al.*, 2003 ▸). In addition to anti­diabetics, metformin shows confirmed benefits against aging (Barzilai *et al.*, 2016 ▸) and various diseases such as polycystic ovary syndrome (Lord *et al.*, 2003 ▸), cancers (Libby *et al.*, 2009 ▸), obesity (Jing *et al.*, 2018 ▸), liver disease (Lin *et al.*, 2000 ▸) and cardiovascular disease (Rena & Lang, 2018 ▸). In recent decades, there has been great inter­est in metformin because of its multiple medical applications and low toxicity. However, metformin also has some disadvantages, such as low bioavailability, incomplete absorption, and gastrointestinal side effects. Gliclazide is an oral sulfonyl­urea anti­diabetic agent that works by stimulating insulin synthesis (Sarkar *et al.*, 2011 ▸). We think that the combination of the two with different mechanisms of action can synergize and result in a potent hypoglycemic effect. In addition, the combination can improve their physico-chemical properties and alleviate the side effects caused by high doses of a single drug.

Introducing sulfonyl into small medical mol­ecules is an important strategy in modifying the mol­ecular structure of drugs. Sulfonyl can provide two hydrogen-bond acceptors, and the introduction of the sulfonyl group can improve the bioactivity of the compound by increasing the hydrogen-bond inter­actions between drug and target. In addition, the sulfonyl group has a relatively stable structure, and the introduction of sulfonyl can block easily metabolizable sites and prolong its time of action, improving its bioavailability, and thereby improving the pharmacokinetic properties of small mol­ecules. In summary, it makes sense to synthesize ion pairs of gliclazide and sulfonyl-modified metformin and investigate its pharmaceutical properties. Herein we report the crystal structure and Hirshfeld surface analysis of the title compound, C_10_H_14_BrN_5_O_2_S, obtained during our efforts to crystallize the ion pair with gliclazide. 
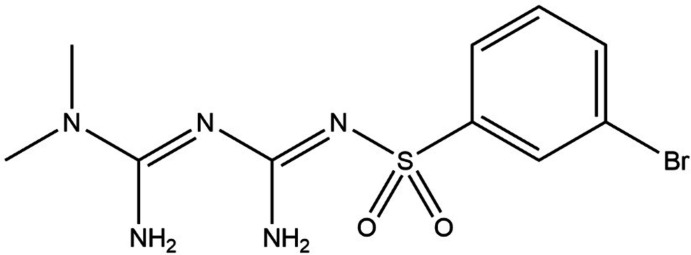


## 2. Structural commentary

**Figure 1:**
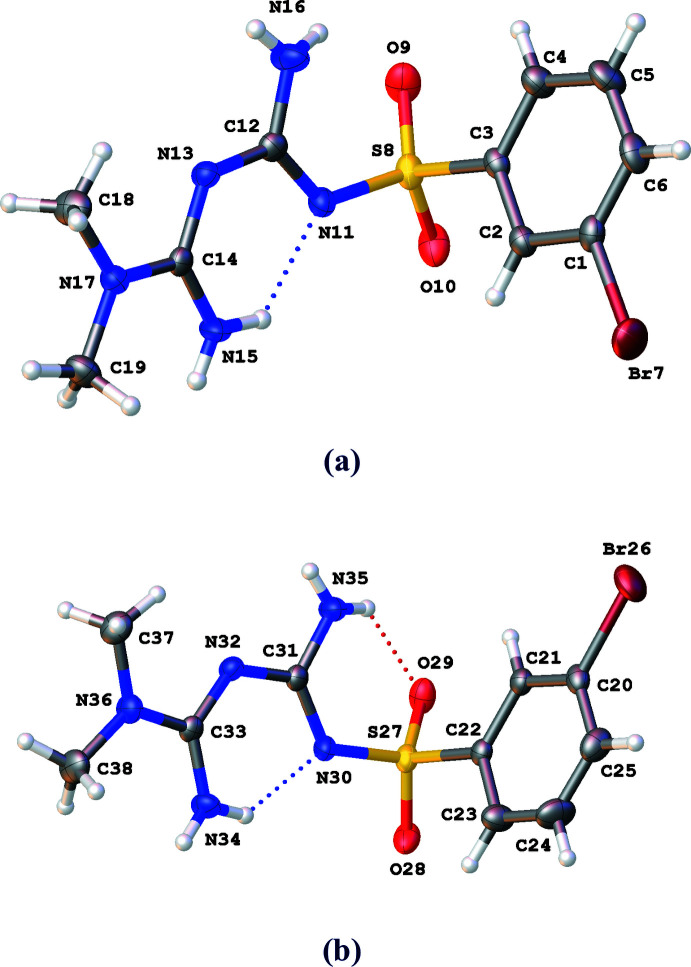
The mol­ecular structure of the two independent mol­ecules (*A* and *B*) of the title compound, showing the atom labelling scheme. Displacement ellipsoids are drawn at the 30% probability level. Only the major component is shown. Intra­molecular inter­actions are shown as dotted lines.

**Figure 2:**
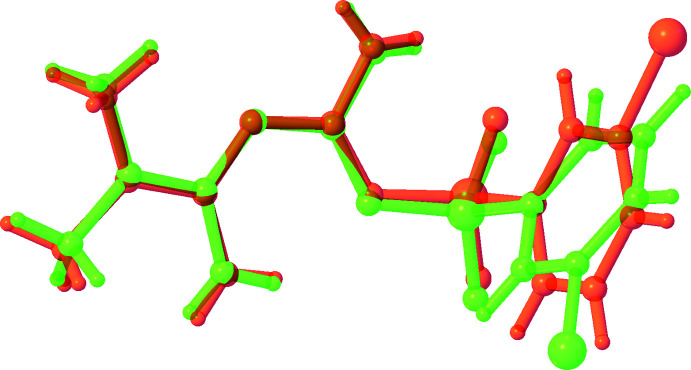
A view of the mol­ecular fit of the *A* (green) and *B* (orange) mol­ecules of the title compound (major component) calculated using the Overlay routine in *OLEX2* (Dolomanov *et al.*, 2009 ▸).

The title compound crystallizes in the triclinic space group *P* 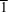 with two mol­ecules (*A* containing S8 and *B* containing S27) in the asymmetric unit (Fig. 1 ▸). Although both mol­ecules have an almost identical conformation, the bromo­phenyl part shows two orientations related by a rotation of 180° (Fig. 2 ▸). The hydrogen atoms involved in the intra­molecular hydrogen bonds N11⋯N15 (mol­ecule *A*) and N30⋯N34 (mol­ecule *B*) are shared by the two nitro­gen atoms with an occupancy of 0.85 (4) at atoms N15 and N34, and 0.15 (4) at atoms N11 and N30. The dihedral angles between the phenyl ring (C1–C6 in *A*, C20–C25 in *B*) and the best plane through the N-containing moiety (N11–C19 in *A* and N30–C38 in *B*) are 87.12 (12) and 96.05 (12)° in *A* and *B*, respectively. Next to the intra­molecular hydrogen bonds N15—H15*B*⋯N11 and N34—H34*B*⋯N30, a short inter­action is present between atoms H16*B* and O9 in *A*, and H35*B* and O9 in *B* (Table 1 ▸).

## 3. Supra­molecular features

**Figure 3:**
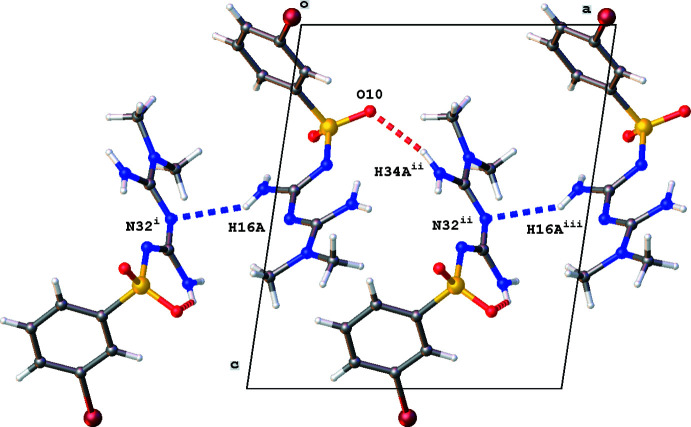
Partial crystal packing of the title compound, showing the chain formation in the *a*-direction. N—H⋯N and N—H⋯O hydrogen bonding are shown as blue and red dashed lines, respectively. Symmetry codes: (i) −*x*, −*y* + 1, −*z* + 1, (ii) −*x* + 1, −*y* + 1, −*z* + 1, (iii) *x* + 1, *y*, *z.*

**Figure 4:**
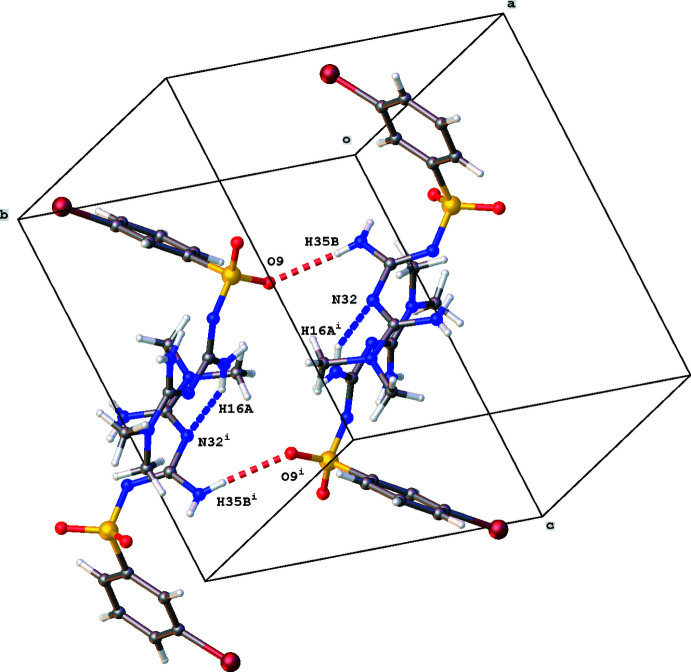
Partial crystal packing of the title compound, showing *R* ^4^_4_(20) ring formation through N—H⋯N and N—H⋯O hydrogen bonding shown as blue and red dashed lines, respectively. Symmetry code: (i) −*x*, −*y* + 1, −*z* + 1*.*

**Figure 5:**
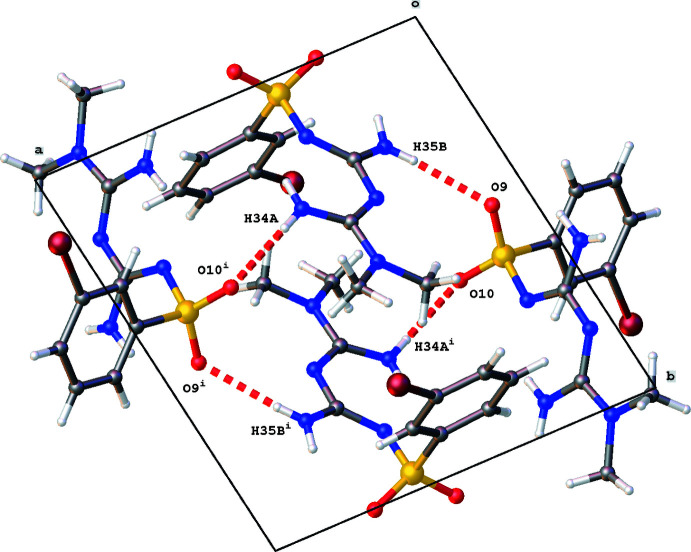
Partial crystal packing of the title compound, showing *R* ^4^_4_(20) ring formation through N—H⋯O hydrogen bonding shown as red dashed lines. Symmetry code: (i) −*x* + 1, −*y* + 1, −*z* + 1*.*

The crystal packing of the title compound is characterized by N—H⋯N, N—H⋯O and π–π inter­actions. The two mol­ecules *A* and *B* in the asymmetric unit are linked by an N35—H35*B*⋯O9 inter­action (Table 1 ▸). Mol­ecule *A* inter­acts with a second mol­ecule *B* by an N16—H16*A*⋯N32(−*x*, −*y* + 1, −*z* + 1) inter­action, while mol­ecule *B* forms an N34—H34*A*⋯O10(−*x* + 1, −*y* + 1, −*z* + 1) hydrogen-bond inter­action (Table 1 ▸). These dimers [graph-set notation *D* ^1^_1_(2); Etter & MacDonald, 1990 ▸] are the building blocks for a three-dimensional network consisting of chains [graph-set notation 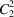 (10)] and rings [graph-set notation 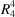 (20)]. A chain running in the *a*-axis direction is formed by subsequent N16⋯N32 and N34⋯O10 inter­actions (Fig. 3 ▸). One ring motif consists of N16⋯N32 and N35 ⋯O9 inter­actions (Fig. 4 ▸), while N34⋯O10 and N35 ⋯O9 inter­actions result in the second ring motif (Fig. 5 ▸).

**Figure 6:**
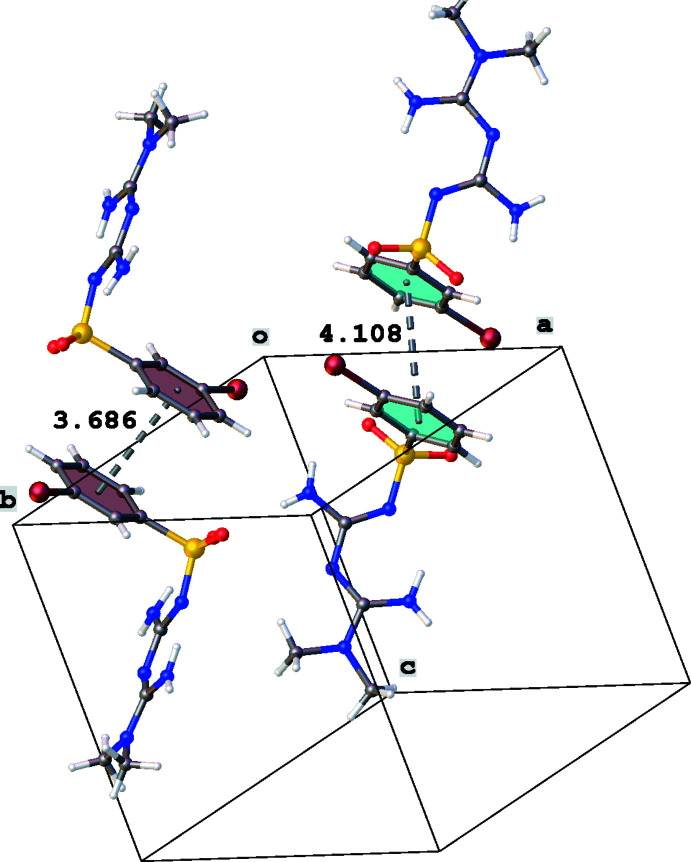
Partial crystal packing of the title compound, showing the π–π stacking between the phenyl rings. Centroid to centroid distances are given in Å.

Further dimer formation is obtained through π–π stacking between the phenyl rings (Fig. 6 ▸). For mol­ecule *A*, the *Cg*1⋯*Cg*1(−*x*, −*y* + 1, −*z*) distance is 3.686 (3) Å and the slippage is 0.650 Å, while for mol­ecule *B* the *Cg*2⋯*Cg*2(−*x* + 1, −*y*, −*z*) distance is 4.1086 (3) Å and the slippage is 1.936 Å (*Cg*1 and *Cg*2 are the centroids of rings C1–C6 and C20–C25, respectively).

**Figure 7:**
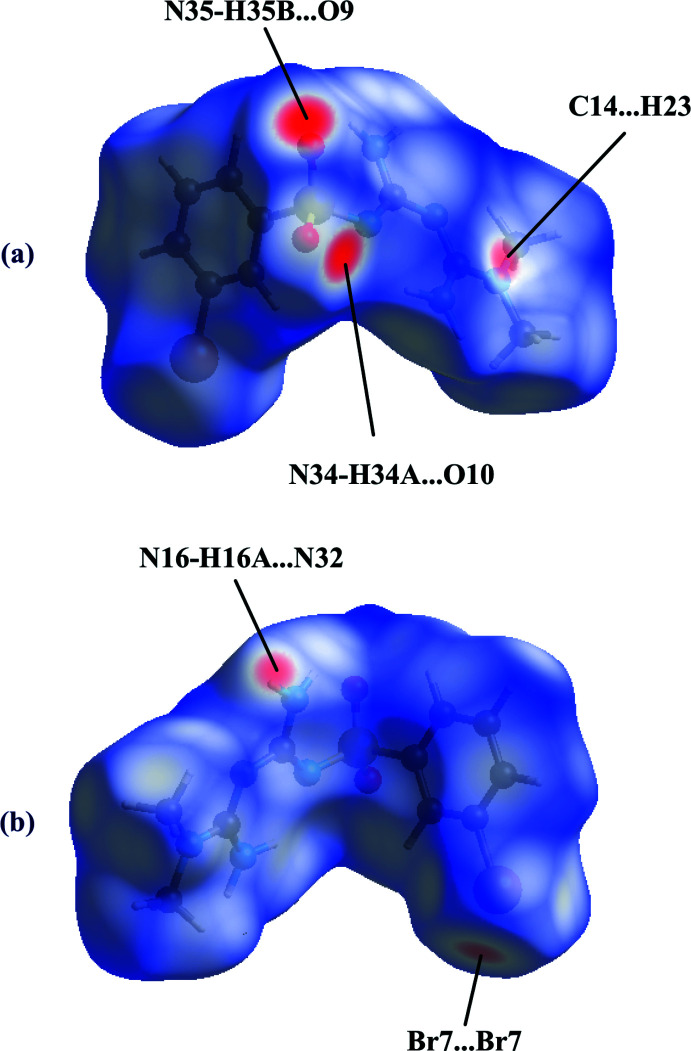
The Hirshfeld surface for mol­ecule *A* mapped over *d*_norm_: (*a*) front view, (*b*) back view.

**Figure 8:**
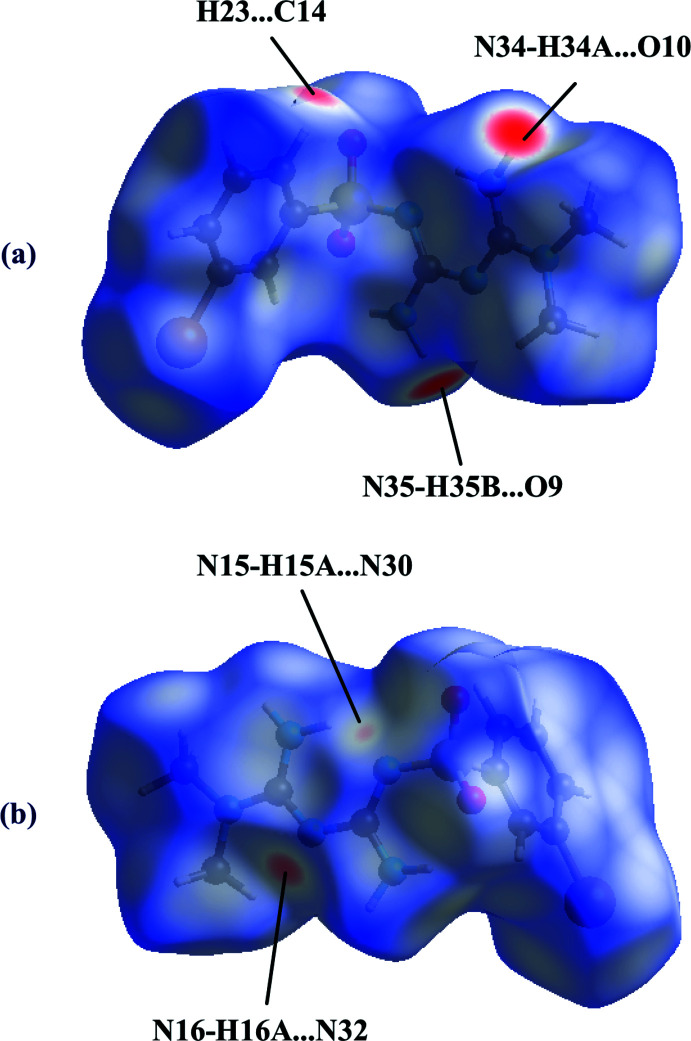
The Hirshfeld surface for mol­ecule *B* mapped over *d*_norm_: (*a*) front view, (*b*) back view.

A Hirshfeld surface analysis was performed, and two-dimensional fingerprint plots were created with *Crystal Explorer21.3* (Spackman *et al.*, 2021 ▸). The Hirshfeld surfaces of mol­ecules *A* and *B* mapped over *d*_norm_ are given in Figs. 7 ▸ and 8 ▸, respectively. The bright-red spots in Fig. 7 ▸ near atoms O9 and O10 are indicative of the N34—H34*A*⋯O10 and N35—H35*B*⋯O9 hydrogen bonds, while the additional faint-red spots illustrate weaker C14⋯H23 (2.66 Å), H16*A*⋯N32 [2.54 (5) Å] and Br⋯Br [3.4165 (10) Å] inter­actions present in the crystal packing. The bright-red spots in Fig. 8 ▸ near atoms N32, H34*A* and H35*B* refer to the N16—H16*A*⋯N32, N34—H34*A*⋯O10 and N35—H35*B*⋯O9 hydrogen bonds, while the additional faint-red spots illustrate weaker H15*A*⋯N30 [2.70 (5) Å] and C14⋯H23 (2.66 Å) inter­actions present in the crystal packing. The relative distributions from the different inter­atomic contacts to the Hirshfeld surfaces are presented in Table 2 ▸. The most significant contributions to the Hirshfeld surface are H⋯H (35.0%, 34.0%), O⋯H/H⋯O (19.2%, 17.7%), H⋯Br/Br⋯H (14.1%, 14.6%), H⋯C/C⋯H (13.1%, 15.0%), and H⋯N/N⋯H (11.5%, 10.5%) contacts (values for mol­ecule *A* and *B*, respectively).

## 4. Database survey

**Figure 9:**
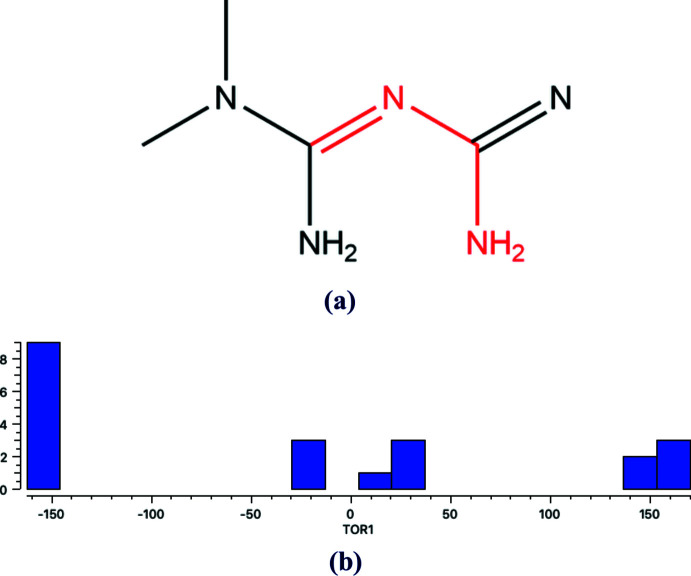
(*a*) Fragment used for search in Cambridge Structural Database, (*b*) histogram of torsion angle TOR1 [shown in red in (*a*)].

A search of the Cambridge Structural Database (CSD, Version 5.43, update of November 2022; Groom *et al.*, 2016 ▸) for the N-containing part of the title compound, as shown in Fig. 9 ▸ *a* resulted in 17 hits [DEXBUF and DEXBUF01 (Nanubolu *et al.*, 2013 ▸), DELKAK (Diniz *et al.*, 2022 ▸), EQUTIV (Olar *et al.*, 2010*a* ▸), EWISAH (Polito-Lucas *et al.*, 2021 ▸), JUMXOH (Bian *et al.*, 2020 ▸), MAXJAA (Sun *et al.*, 2022 ▸), NAKWAB (Manjunatha *et al.*, 2020 ▸), NICCEJ (Satyanarayana Reddy *et al.*, 2013 ▸), NUPXED (Dong *et al.*, 2015 ▸), OJOSUC (Olar *et al.*, 2010*b* ▸), OJOSUC01 (Wei *et al.*, 2014 ▸), QILBOF (Sánchez-Lara *et al.*, 2018 ▸), ROLFUW (Jia *et al.*, 2019 ▸), UKODUW01 (Feng *et al.*, 2021 ▸), WIBSIJ (Lemoine *et al.*, 1994 ▸), YEJVOC (Jiang *et al.*, 2022 ▸); for more details, see the supporting information]. In contrast to the title compound, all 17 compounds bear a positve charge. The histogram of the torsion angle TOR1 illustrates that the majority of these fragments are non-planar (Fig. 9 ▸ *b*). For the title compound, this torsion angle is −177.5 (4) and −171.8 (4)° in *A* and *B*, respectively.

## 5. Synthesis and crystallization

**Figure 10:**
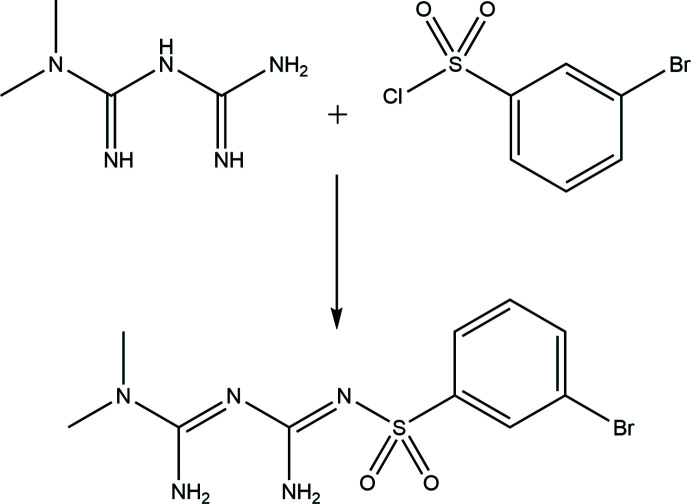
Reaction scheme for the synthesis of the title compound.

The reaction scheme to synthesize the title compound is given in Fig. 10 ▸.

Metformin hydro­chloride (662.5 mg, 4.0 mmol) was dissolved in 1*M* sodium hydroxide solution (320 ml, 8.0 mmol). The mixture was stirred for 30 min at room temperature. After the reaction was complete, water was removed under reduced pressure and the residue was dissolved in cold anhydrous methanol. The sodium chloride was filtered off and the filtrate was evaporated under reduced pressure to obtain basic metformin.

The basic metformin (258.2 mg, 2.0 mmol) and 3-bromo­benzene­sulfonyl chloride (144 µL, 1.0 mmol) were dissolved in 6 mL of anhydrous di­chloro­methane and stirred under a nitro­gen atmosphere for 3 h at room temperature. The solvent was removed on a rotary evaporator and the residue was purified by column chromatography (eluent: MeOH: CH_2_Cl_2_ = 1:10) to obtain the title compound as a colourless solid.

To obtain its hydro­chloride salt, the title compound was dissolved in ethanol and stirred at room temperature. An ethanol solution of hydro­chloric acid was added dropwise until pH = 2 and the reaction was followed by TLC. After completion of the reaction, the solvent was removed under reduced pressure to obtain the hydro­chloride salt.

The hydro­chloride salt (76.7 mg, 0.2 mmol) and sodium gliclazide (69.3 mg, 0.2 mmol) were dissolved in 5 mL of acetone and stirred overnight at room temperature. The solvent was removed under reduced pressure and a light-yellow solid was obtained, which was expected to be the sulfonyl­urea salt of the title compound.

Cuboid-shaped colourless crystals were grown in an NMR tube by slow evaporation over two weeks using deuterated chloro­form as solvent. However, the grown crystals consist of the title compound and not of its sulfonyl­urea salt.

NMR spectra of the title compound were recorded on a 400 MHz NMR spectrometer: ^1^H NMR (400 MHz, CDCl_3_) δ 8.03 (*t*, *J* = 1.7 Hz, 1H, phen­yl), 7.90–7.76 (*m*, 1H, phen­yl), 7.64–7.57 (*m*, 1H, phen­yl), 7.56–7.22 (*m*, 3H, phenyl and NH_2_), 7.06 (*s*, 1H, NH_2_), 5.19 (*s*, 1H, NH_2_), 2.99 (*s*, 6H, CH_3_). ^13^C NMR (101 MHz, CDCl_3_) δ 160.29 (*s*), 158.59 (*s*), 145.65 (*s*), 134.41 (*s*), 130.20 (*s*), 129.13 (*s*), 124.70 (*s*), 122.54 (*s*), 37.00 (*s*).

## 6. Refinement

Crystal data, data collection and structure refinement details are summarized in Table 3 ▸. All hydrogen atoms bound to carbon were placed at idealized positions and refined using a riding model, with *U*_iso_(H) values assigned as 1.2*U*_eq_ or 1.5*U*_eq_ (methyl only) of the parent atoms, with C—H distances of 0.93 (aromatic) and 0.96 Å (meth­yl). The hydrogen atoms bound to nitro­gen were located in a difference-Fourier map and refined freely with *U*_iso_(H) values assigned as 1.2*U*_eq_ of the parent atoms. The occupancy factors of hydrogen atoms H11 and H15*B* (mol­ecule *A*), and H30 and H34*B* (mol­ecule *B*) involved in intra­molecular hydrogen bonds converged during refinement to 0.85 (4) for H15*B* and H34*B*, and 0.15 (4) for H11 and H30.

## Supplementary Material

Crystal structure: contains datablock(s) I. DOI: 10.1107/S2056989023002165/pk2683sup1.cif

Structure factors: contains datablock(s) I. DOI: 10.1107/S2056989023002165/pk2683Isup2.hkl

CSD survey results. DOI: 10.1107/S2056989023002165/pk2683sup3.pdf

Supporting information file. DOI: 10.1107/S2056989023002165/pk2683Isup4.cml

CCDC reference: 2246792

Additional supporting information:  crystallographic information; 3D view; checkCIF report

## Figures and Tables

**Table 1:** Hydrogen-bond geometry (Å, °)

*D*—H⋯*A*	*D*—H	H⋯*A*	*D*⋯*A*	*D*—H⋯*A*
N15—H15*B*⋯N11	0.86 (6)	2.02 (6)	2.655 (6)	130 (5)
N16—H16*B*⋯O9	0.83 (6)	2.19 (5)	2.830 (6)	134 (5)
N34—H34*B*⋯N30	0.86 (6)	2.02 (6)	2.696 (5)	135 (5)
N35—H35*A*⋯O29	0.83 (5)	2.18 (5)	2.823 (6)	136 (4)
N35—H35*B*⋯O9	0.78 (5)	2.27 (5)	3.049 (6)	175 (5)
N16—H16*A*⋯N32^i^	0.81 (5)	2.54 (5)	3.225 (6)	144 (4)
N34—H34*A*⋯O10^ii^	0.85 (5)	2.23 (5)	3.063 (5)	165 (4)

Symmetry codes: (i) 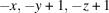 ; (ii) 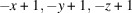 .

**Table 2:** Percentage contributions of inter­atomic contacts to the Hirshfeld surfaces for mol­ecules *A* and *B*

Contact	Mol­ecule *A*	Mol­ecule *B*
C⋯C	3.2	2.4
C⋯H/H⋯C	13.1	15.0
H⋯H	35.0	34.0
Br⋯C/C⋯Br	0.2	2.1
Br⋯H/H⋯Br	14.1	14.6
Br⋯Br	1.7	0.0
S⋯C/C⋯S	0.0	0.0
S⋯H/H⋯S	0.1	0.1
S⋯Br/Br⋯S	0.0	0.0
S⋯S	0.0	0.0
O⋯C/C⋯O	0.4	0.0
O⋯H/H⋯O	19.2	17.7
O⋯Br/Br⋯O	0.8	2.9
O⋯S/S⋯O	0.0	0.0
O⋯O	0.1	0.1
N⋯C/C⋯N	0.3	0.2
N⋯H/H⋯N	11.5	10.5
N⋯Br/Br⋯N	0.0	0.0
N⋯S/S⋯N	0.0	0.0
N⋯O/O⋯N	0.0	0.0
N⋯N	0.2	0.4

**Table 3:** Experimental details

Crystal data	
Chemical formula	C_10_H_14_BrN_5_O_2_S
*M*_r_	348.23
Crystal system, space group	Triclinic, *P* 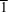
Temperature (K)	294
*a*, *b*, *c* (Å)	10.3839 (5), 11.3296 (6), 12.3477 (7)
α, β, γ (°)	103.393 (5), 96.380 (4), 97.934 (4)
*V* (Å^3^)	1384.10 (13)
*Z*	4
Radiation type	Mo *K*α
μ (mm^−1^)	3.13
Crystal size (mm)	0.6 × 0.3 × 0.3

Data collection	
Diffractometer	SuperNova, Single source at offset/far, Eos
Absorption correction	Multi-scan (*CrysAlis PRO*; Rigaku OD, 2022 ▸)
*T*_min_, *T*_max_	0.417, 1.000
No. of measured, independent and observed [*I* > 2σ(*I*)] reflections	16803, 5658, 3549
*R*_int_	0.051
(sin θ/λ)_max_ (Å^−1^)	0.625

Refinement	
*R*[*F* ^2^ > 2σ(*F* ^2^)], *wR*(*F* ^2^), *S*	0.053, 0.117, 1.02
No. of reflections	5658
No. of parameters	372
H-atom treatment	H atoms treated by a mixture of independent and constrained refinement
Δρ_max_, Δρ_min_ (e Å^−3^)	1.05, −0.88

Computer programs: *CrysAlis PRO* (Rigaku OD, 2022 ▸), *SHELXT2014/5* (Sheldrick, 2015*a* ▸), *SHELXL2016/4* (Sheldrick, 2015*b* ▸) and *OLEX2* (Dolomanov *et al.*, 2009 ▸).

## supplementary crystallographic information

### Crystal data

**Table:**

C_10_H_14_BrN_5_O_2_S	*Z* = 4
*M**_r_* = 348.23	*F*(000) = 704
Triclinic, *P*1	*D*_x_ = 1.671 Mg m^−^^3^
*a* = 10.3839 (5) Å	Mo *K*α radiation, λ = 0.71073 Å
*b* = 11.3296 (6) Å	Cell parameters from 4121 reflections
*c* = 12.3477 (7) Å	θ = 2.8–25.9°
α = 103.393 (5)°	µ = 3.13 mm^−^^1^
β = 96.380 (4)°	*T* = 294 K
γ = 97.934 (4)°	Block, colourless
*V* = 1384.10 (13) Å^3^	0.6 × 0.3 × 0.3 mm

### Data collection

**Table:**

SuperNova, Single source at offset/far, Eos diffractometer	5658 independent reflections
Radiation source: micro-focus sealed X-ray tube, SuperNova (Mo) X-ray Source	3549 reflections with *I* > 2σ(*I*)
Mirror monochromator	*R*_int_ = 0.051
Detector resolution: 15.9631 pixels mm^-1^	θ_max_ = 26.4°, θ_min_ = 2.5°
ω scans	*h* = −12→12
Absorption correction: multi-scan (CrysAlisPro; Rigaku OD, 2022)	*k* = −14→14
*T*_min_ = 0.417, *T*_max_ = 1.000	*l* = −15→15
16803 measured reflections	

### Refinement

**Table:**

Refinement on *F*^2^	Primary atom site location: dual
Least-squares matrix: full	Hydrogen site location: mixed
*R*[*F*^2^ > 2σ(*F*^2^)] = 0.053	H atoms treated by a mixture of independent and constrained refinement
*wR*(*F*^2^) = 0.117	*w* = 1/[σ^2^(*F*_o_^2^) + (0.035*P*)^2^ + 1.4413*P*] where *P* = (*F*_o_^2^ + 2*F*_c_^2^)/3
*S* = 1.02	(Δ/σ)_max_ < 0.001
5658 reflections	Δρ_max_ = 1.05 e Å^−^^3^
372 parameters	Δρ_min_ = −0.88 e Å^−^^3^
0 restraints	

### Special details

**Table:**

Geometry. All esds (except the esd in the dihedral angle between two l.s. planes) are estimated using the full covariance matrix. The cell esds are taken into account individually in the estimation of esds in distances, angles and torsion angles; correlations between esds in cell parameters are only used when they are defined by crystal symmetry. An approximate (isotropic) treatment of cell esds is used for estimating esds involving l.s. planes.

### Fractional atomic coordinates and isotropic or equivalent isotropic displacement parameters (Å^2^)

**Table:**

	*x*	*y*	*z*	*U*_iso_*/*U*_eq_	Occ. (<1)
C1	−0.0600 (5)	0.7080 (4)	0.0460 (3)	0.0392 (11)	
C2	0.0355 (4)	0.6995 (4)	0.1296 (3)	0.0357 (11)	
H2	0.111511	0.758247	0.153617	0.043*	
C3	0.0135 (4)	0.5996 (4)	0.1764 (3)	0.0318 (10)	
C4	−0.0994 (5)	0.5123 (4)	0.1403 (4)	0.0442 (12)	
H4	−0.112981	0.445965	0.172576	0.053*	
C5	−0.1915 (5)	0.5246 (5)	0.0562 (4)	0.0530 (13)	
H5	−0.267409	0.465854	0.031437	0.064*	
C6	−0.1727 (5)	0.6219 (5)	0.0087 (4)	0.0469 (13)	
H6	−0.235200	0.629864	−0.048086	0.056*	
Br7	−0.03344 (6)	0.84386 (6)	−0.02036 (5)	0.0661 (2)	
S8	0.13699 (11)	0.58384 (11)	0.28223 (9)	0.0367 (3)	
O9	0.0932 (3)	0.4667 (3)	0.3068 (3)	0.0508 (9)	
O10	0.2597 (3)	0.5961 (3)	0.2382 (3)	0.0503 (9)	
N11	0.1473 (3)	0.6992 (3)	0.3852 (3)	0.0347 (9)	
H11	0.214239	0.756936	0.396878	0.042*	0.15 (4)
C12	0.0568 (4)	0.7124 (4)	0.4561 (3)	0.0337 (10)	
N13	0.0563 (3)	0.8161 (3)	0.5341 (3)	0.0324 (8)	
C14	0.1437 (4)	0.9194 (4)	0.5488 (3)	0.0308 (10)	
N15	0.2461 (4)	0.9308 (4)	0.4932 (4)	0.0435 (11)	
H15A	0.301 (5)	0.992 (5)	0.506 (4)	0.052*	
H15B	0.253 (5)	0.871 (5)	0.438 (5)	0.052*	0.85 (4)
N16	−0.0418 (5)	0.6209 (4)	0.4518 (4)	0.0513 (12)	
H16A	−0.088 (5)	0.634 (5)	0.500 (4)	0.062*	
H16B	−0.035 (5)	0.549 (5)	0.422 (4)	0.062*	
N17	0.1270 (3)	1.0179 (3)	0.6273 (3)	0.0364 (9)	
C18	0.0186 (5)	1.0110 (5)	0.6928 (4)	0.0543 (14)	
H18A	−0.035482	0.931202	0.666556	0.081*	
H18B	−0.033126	1.073065	0.684068	0.081*	
H18C	0.053284	1.024212	0.770841	0.081*	
C19	0.2135 (5)	1.1361 (4)	0.6488 (4)	0.0494 (13)	
H19A	0.303190	1.125183	0.663859	0.074*	
H19B	0.192002	1.192125	0.712777	0.074*	
H19C	0.202254	1.169086	0.584087	0.074*	
C20	0.5361 (5)	0.1808 (4)	0.0442 (4)	0.0398 (12)	
C21	0.4466 (4)	0.1344 (4)	0.1054 (3)	0.0359 (11)	
H21	0.356697	0.128306	0.084312	0.043*	
C22	0.4950 (4)	0.0973 (4)	0.1993 (3)	0.0311 (10)	
C23	0.6286 (5)	0.1067 (4)	0.2308 (4)	0.0484 (13)	
H23	0.660272	0.083478	0.294840	0.058*	
C24	0.7145 (5)	0.1507 (5)	0.1663 (5)	0.0645 (16)	
H24	0.804515	0.155822	0.186400	0.077*	
C25	0.6690 (5)	0.1871 (5)	0.0730 (5)	0.0527 (14)	
H25	0.727602	0.215887	0.029400	0.063*	
Br26	0.47490 (7)	0.23897 (6)	−0.08045 (5)	0.0725 (2)	
S27	0.38479 (12)	0.04014 (10)	0.28332 (9)	0.0356 (3)	
O28	0.4452 (3)	−0.0477 (3)	0.3299 (3)	0.0496 (9)	
O29	0.2591 (3)	−0.0032 (3)	0.2118 (3)	0.0467 (8)	
N30	0.3838 (3)	0.1539 (3)	0.3879 (3)	0.0343 (9)	
H30	0.428755	0.156416	0.451519	0.041*	0.15 (4)
C31	0.3160 (4)	0.2461 (4)	0.3813 (3)	0.0319 (10)	
N32	0.3220 (3)	0.3472 (3)	0.4660 (3)	0.0330 (8)	
C33	0.4065 (4)	0.3742 (4)	0.5619 (3)	0.0307 (10)	
N34	0.4890 (4)	0.3022 (4)	0.5916 (3)	0.0414 (10)	
H34A	0.550 (5)	0.327 (4)	0.648 (4)	0.050*	
H34B	0.486 (5)	0.234 (5)	0.543 (5)	0.050*	0.85 (4)
N35	0.2361 (4)	0.2468 (4)	0.2884 (3)	0.0434 (11)	
H35A	0.213 (5)	0.182 (4)	0.239 (4)	0.052*	
H35B	0.196 (5)	0.301 (4)	0.295 (4)	0.052*	
N36	0.4036 (4)	0.4825 (3)	0.6346 (3)	0.0418 (10)	
C37	0.3317 (5)	0.5744 (4)	0.6032 (4)	0.0510 (13)	
H37A	0.290176	0.544562	0.526308	0.076*	
H37B	0.391734	0.649473	0.611375	0.076*	
H37C	0.266048	0.589615	0.651189	0.076*	
C38	0.4763 (5)	0.5172 (5)	0.7476 (4)	0.0593 (15)	
H38A	0.473851	0.445938	0.777398	0.089*	
H38B	0.437222	0.578041	0.794622	0.089*	
H38C	0.565894	0.550465	0.745539	0.089*	

### Atomic displacement parameters (Å^2^)

**Table:**

	*U*^11^	*U*^22^	*U*^33^	*U*^12^	*U*^13^	*U*^23^
C1	0.048 (3)	0.042 (3)	0.027 (2)	0.016 (3)	0.005 (2)	0.003 (2)
C2	0.035 (3)	0.036 (3)	0.033 (2)	0.005 (2)	0.002 (2)	0.002 (2)
C3	0.028 (3)	0.033 (3)	0.030 (2)	0.008 (2)	0.0028 (19)	−0.0012 (19)
C4	0.040 (3)	0.039 (3)	0.049 (3)	0.003 (2)	−0.001 (2)	0.009 (2)
C5	0.039 (3)	0.047 (3)	0.061 (3)	−0.003 (3)	−0.010 (3)	0.005 (3)
C6	0.037 (3)	0.057 (3)	0.040 (3)	0.014 (3)	−0.008 (2)	0.001 (3)
Br7	0.0806 (4)	0.0701 (4)	0.0497 (3)	0.0082 (3)	−0.0065 (3)	0.0297 (3)
S8	0.0382 (7)	0.0386 (7)	0.0329 (6)	0.0165 (6)	0.0017 (5)	0.0036 (5)
O9	0.070 (2)	0.0349 (19)	0.051 (2)	0.0233 (18)	0.0047 (18)	0.0108 (16)
O10	0.0370 (19)	0.070 (2)	0.0405 (18)	0.0223 (18)	0.0074 (15)	−0.0008 (17)
N11	0.033 (2)	0.038 (2)	0.0307 (19)	0.0037 (18)	0.0064 (17)	0.0028 (17)
C12	0.036 (3)	0.034 (3)	0.033 (2)	0.008 (2)	0.003 (2)	0.012 (2)
N13	0.035 (2)	0.030 (2)	0.0323 (19)	0.0048 (18)	0.0110 (17)	0.0060 (17)
C14	0.034 (3)	0.035 (3)	0.024 (2)	0.008 (2)	0.0017 (19)	0.0094 (19)
N15	0.040 (3)	0.041 (3)	0.042 (2)	−0.012 (2)	0.013 (2)	0.003 (2)
N16	0.060 (3)	0.031 (2)	0.061 (3)	−0.001 (2)	0.028 (2)	0.002 (2)
N17	0.036 (2)	0.035 (2)	0.035 (2)	0.0012 (19)	0.0055 (18)	0.0046 (17)
C18	0.057 (4)	0.048 (3)	0.058 (3)	0.012 (3)	0.028 (3)	0.003 (3)
C19	0.052 (3)	0.034 (3)	0.056 (3)	0.000 (3)	0.002 (3)	0.005 (2)
C20	0.058 (3)	0.030 (3)	0.033 (2)	0.011 (2)	0.013 (2)	0.006 (2)
C21	0.039 (3)	0.035 (3)	0.032 (2)	0.010 (2)	0.008 (2)	0.003 (2)
C22	0.034 (3)	0.027 (2)	0.029 (2)	0.005 (2)	0.007 (2)	0.0001 (18)
C23	0.043 (3)	0.054 (3)	0.054 (3)	0.010 (3)	0.007 (3)	0.025 (3)
C24	0.036 (3)	0.079 (4)	0.089 (4)	0.013 (3)	0.015 (3)	0.037 (4)
C25	0.051 (4)	0.047 (3)	0.068 (4)	0.009 (3)	0.026 (3)	0.021 (3)
Br26	0.1075 (5)	0.0769 (5)	0.0443 (3)	0.0238 (4)	0.0172 (3)	0.0299 (3)
S27	0.0461 (7)	0.0289 (6)	0.0326 (6)	0.0081 (6)	0.0128 (5)	0.0053 (5)
O28	0.075 (2)	0.0375 (19)	0.0488 (19)	0.0247 (18)	0.0270 (18)	0.0193 (16)
O29	0.043 (2)	0.045 (2)	0.0437 (19)	−0.0034 (16)	0.0109 (16)	−0.0031 (16)
N30	0.044 (2)	0.032 (2)	0.0283 (18)	0.0132 (18)	0.0067 (17)	0.0045 (16)
C31	0.032 (3)	0.033 (3)	0.031 (2)	0.005 (2)	0.010 (2)	0.008 (2)
N32	0.032 (2)	0.031 (2)	0.035 (2)	0.0084 (17)	0.0030 (17)	0.0033 (16)
C33	0.024 (2)	0.030 (2)	0.035 (2)	0.000 (2)	0.008 (2)	0.006 (2)
N34	0.039 (3)	0.040 (3)	0.037 (2)	0.008 (2)	−0.0092 (19)	0.001 (2)
N35	0.049 (3)	0.043 (3)	0.037 (2)	0.019 (2)	−0.002 (2)	0.0046 (19)
N36	0.049 (2)	0.034 (2)	0.036 (2)	0.007 (2)	0.0013 (19)	−0.0022 (18)
C37	0.056 (3)	0.032 (3)	0.060 (3)	0.006 (3)	0.008 (3)	0.002 (2)
C38	0.063 (4)	0.054 (3)	0.046 (3)	0.013 (3)	−0.005 (3)	−0.014 (3)

### Geometric parameters (Å, º)

**Table:**

C1—C2	1.381 (6)	C20—C21	1.385 (6)
C1—C6	1.374 (6)	C20—C25	1.374 (7)
C1—Br7	1.906 (5)	C20—Br26	1.890 (4)
C2—H2	0.9300	C21—H21	0.9300
C2—C3	1.389 (6)	C21—C22	1.388 (6)
C3—C4	1.382 (6)	C22—C23	1.381 (6)
C3—S8	1.784 (4)	C22—S27	1.782 (4)
C4—H4	0.9300	C23—H23	0.9300
C4—C5	1.377 (6)	C23—C24	1.378 (7)
C5—H5	0.9300	C24—H24	0.9300
C5—C6	1.366 (7)	C24—C25	1.369 (7)
C6—H6	0.9300	C25—H25	0.9300
S8—O9	1.453 (3)	S27—O28	1.439 (3)
S8—O10	1.441 (3)	S27—O29	1.446 (3)
S8—N11	1.581 (4)	S27—N30	1.602 (3)
N11—H11	0.8600	N30—H30	0.8600
N11—C12	1.354 (5)	N30—C31	1.350 (5)
C12—N13	1.336 (5)	C31—N32	1.349 (5)
C12—N16	1.340 (6)	C31—N35	1.342 (6)
N13—C14	1.341 (5)	N32—C33	1.340 (5)
C14—N15	1.336 (5)	C33—N34	1.339 (5)
C14—N17	1.343 (5)	C33—N36	1.349 (5)
N15—H15A	0.80 (5)	N34—H34A	0.85 (5)
N15—H15B	0.86 (6)	N34—H34B	0.85 (6)
N16—H16A	0.80 (5)	N35—H35A	0.83 (5)
N16—H16B	0.83 (5)	N35—H35B	0.78 (5)
N17—C18	1.461 (5)	N36—C37	1.459 (6)
N17—C19	1.457 (6)	N36—C38	1.449 (6)
C18—H18A	0.9600	C37—H37A	0.9600
C18—H18B	0.9600	C37—H37B	0.9600
C18—H18C	0.9600	C37—H37C	0.9600
C19—H19A	0.9600	C38—H38A	0.9600
C19—H19B	0.9600	C38—H38B	0.9600
C19—H19C	0.9600	C38—H38C	0.9600
			
C2—C1—Br7	118.5 (4)	C21—C20—Br26	119.5 (4)
C6—C1—C2	122.4 (4)	C25—C20—C21	121.3 (4)
C6—C1—Br7	119.1 (3)	C25—C20—Br26	119.1 (4)
C1—C2—H2	121.4	C20—C21—H21	120.9
C1—C2—C3	117.2 (4)	C20—C21—C22	118.1 (4)
C3—C2—H2	121.4	C22—C21—H21	120.9
C2—C3—S8	118.3 (3)	C21—C22—S27	120.2 (3)
C4—C3—C2	121.2 (4)	C23—C22—C21	120.9 (4)
C4—C3—S8	120.4 (3)	C23—C22—S27	118.9 (3)
C3—C4—H4	120.3	C22—C23—H23	120.3
C5—C4—C3	119.3 (5)	C24—C23—C22	119.3 (5)
C5—C4—H4	120.3	C24—C23—H23	120.3
C4—C5—H5	119.6	C23—C24—H24	119.6
C6—C5—C4	120.8 (5)	C25—C24—C23	120.8 (5)
C6—C5—H5	119.6	C25—C24—H24	119.6
C1—C6—H6	120.5	C20—C25—H25	120.3
C5—C6—C1	119.0 (4)	C24—C25—C20	119.5 (5)
C5—C6—H6	120.5	C24—C25—H25	120.3
O9—S8—C3	105.9 (2)	O28—S27—C22	106.94 (19)
O9—S8—N11	114.05 (19)	O28—S27—O29	117.7 (2)
O10—S8—C3	106.56 (19)	O28—S27—N30	105.69 (19)
O10—S8—O9	116.50 (19)	O29—S27—C22	106.34 (19)
O10—S8—N11	106.7 (2)	O29—S27—N30	113.73 (18)
N11—S8—C3	106.42 (19)	N30—S27—C22	105.63 (19)
S8—N11—H11	118.1	S27—N30—H30	118.3
C12—N11—S8	123.7 (3)	C31—N30—S27	123.3 (3)
C12—N11—H11	118.1	C31—N30—H30	118.3
N13—C12—N11	123.7 (4)	N32—C31—N30	124.1 (4)
N13—C12—N16	114.6 (4)	N35—C31—N30	123.0 (4)
N16—C12—N11	121.7 (4)	N35—C31—N32	112.9 (4)
C12—N13—C14	123.6 (4)	C33—N32—C31	123.7 (4)
N13—C14—N17	116.7 (4)	N32—C33—N36	115.6 (4)
N15—C14—N13	125.2 (4)	N34—C33—N32	125.8 (4)
N15—C14—N17	118.1 (4)	N34—C33—N36	118.6 (4)
C14—N15—H15A	124 (4)	C33—N34—H34A	123 (3)
C14—N15—H15B	119 (4)	C33—N34—H34B	115 (4)
H15A—N15—H15B	117 (5)	H34A—N34—H34B	121 (5)
C12—N16—H16A	116 (4)	C31—N35—H35A	119 (3)
C12—N16—H16B	119 (4)	C31—N35—H35B	115 (4)
H16A—N16—H16B	120 (5)	H35A—N35—H35B	123 (5)
C14—N17—C18	120.9 (4)	C33—N36—C37	122.3 (4)
C14—N17—C19	121.8 (4)	C33—N36—C38	122.0 (4)
C19—N17—C18	117.3 (4)	C38—N36—C37	115.7 (4)
N17—C18—H18A	109.5	N36—C37—H37A	109.5
N17—C18—H18B	109.5	N36—C37—H37B	109.5
N17—C18—H18C	109.5	N36—C37—H37C	109.5
H18A—C18—H18B	109.5	H37A—C37—H37B	109.5
H18A—C18—H18C	109.5	H37A—C37—H37C	109.5
H18B—C18—H18C	109.5	H37B—C37—H37C	109.5
N17—C19—H19A	109.5	N36—C38—H38A	109.5
N17—C19—H19B	109.5	N36—C38—H38B	109.5
N17—C19—H19C	109.5	N36—C38—H38C	109.5
H19A—C19—H19B	109.5	H38A—C38—H38B	109.5
H19A—C19—H19C	109.5	H38A—C38—H38C	109.5
H19B—C19—H19C	109.5	H38B—C38—H38C	109.5
			
C1—C2—C3—C4	0.1 (6)	C20—C21—C22—C23	0.1 (6)
C1—C2—C3—S8	178.5 (3)	C20—C21—C22—S27	178.6 (3)
C2—C1—C6—C5	0.3 (7)	C21—C20—C25—C24	−2.2 (8)
C2—C3—C4—C5	0.2 (6)	C21—C22—C23—C24	−1.6 (7)
C2—C3—S8—O9	−174.6 (3)	C21—C22—S27—O28	149.4 (3)
C2—C3—S8—O10	−49.9 (4)	C21—C22—S27—O29	22.8 (4)
C2—C3—S8—N11	63.6 (4)	C21—C22—S27—N30	−98.3 (4)
C3—C4—C5—C6	−0.3 (7)	C22—C23—C24—C25	1.1 (8)
C3—S8—N11—C12	74.5 (4)	C22—S27—N30—C31	78.0 (4)
C4—C3—S8—O9	3.7 (4)	C23—C22—S27—O28	−32.1 (4)
C4—C3—S8—O10	128.4 (3)	C23—C22—S27—O29	−158.7 (4)
C4—C3—S8—N11	−118.0 (3)	C23—C22—S27—N30	80.1 (4)
C4—C5—C6—C1	0.0 (7)	C23—C24—C25—C20	0.7 (8)
C6—C1—C2—C3	−0.4 (6)	C25—C20—C21—C22	1.8 (7)
Br7—C1—C2—C3	180.0 (3)	Br26—C20—C21—C22	−177.3 (3)
Br7—C1—C6—C5	179.9 (4)	Br26—C20—C25—C24	176.9 (4)
S8—C3—C4—C5	−178.1 (4)	S27—C22—C23—C24	180.0 (4)
S8—N11—C12—N13	−171.1 (3)	S27—N30—C31—N32	−174.7 (3)
S8—N11—C12—N16	7.9 (6)	S27—N30—C31—N35	4.6 (6)
O9—S8—N11—C12	−41.9 (4)	O28—S27—N30—C31	−168.8 (3)
O10—S8—N11—C12	−172.0 (3)	O29—S27—N30—C31	−38.2 (4)
N11—C12—N13—C14	1.6 (6)	N30—C31—N32—C33	7.6 (7)
C12—N13—C14—N15	−4.1 (7)	C31—N32—C33—N34	−5.4 (7)
C12—N13—C14—N17	177.1 (4)	C31—N32—C33—N36	176.4 (4)
N13—C14—N17—C18	0.1 (6)	N32—C33—N36—C37	−10.9 (6)
N13—C14—N17—C19	−178.3 (4)	N32—C33—N36—C38	171.7 (4)
N15—C14—N17—C18	−178.7 (4)	N34—C33—N36—C37	170.8 (4)
N15—C14—N17—C19	2.8 (6)	N34—C33—N36—C38	−6.6 (6)
N16—C12—N13—C14	−177.5 (4)	N35—C31—N32—C33	−171.8 (4)

### Hydrogen-bond geometry (Å, º)

**Table:**

*D*—H···*A*	*D*—H	H···*A*	*D*···*A*	*D*—H···*A*
N15—H15*B*···N11	0.86 (6)	2.02 (6)	2.655 (6)	130 (5)
N16—H16*B*···O9	0.83 (6)	2.19 (5)	2.830 (6)	134 (5)
N34—H34*B*···N30	0.86 (6)	2.02 (6)	2.696 (5)	135 (5)
N35—H35*A*···O29	0.83 (5)	2.18 (5)	2.823 (6)	136 (4)
N35—H35*B*···O9	0.78 (5)	2.27 (5)	3.049 (6)	175 (5)
N16—H16*A*···N32^i^	0.81 (5)	2.54 (5)	3.225 (6)	144 (4)
N34—H34*A*···O10^ii^	0.85 (5)	2.23 (5)	3.063 (5)	165 (4)

Symmetry codes: (i) −*x*, −*y*+1, −*z*+1; (ii) −*x*+1, −*y*+1, −*z*+1.
