# Supplementary material for: Crystal structure and Hirshfeld surface analysis of N-{N-[amino­(di­methyl­amino)­meth­yl]carbamimido­yl}-3-bromo­benzene­sulfonamide
Source: Acta Crystallogr E Crystallogr Commun. 2023 Mar 21;79(Pt 4):367–72. doi: 10.1107/S2056989023002165 (PMC10088321; doi:10.1107/S2056989023002165)

Search Overview

Search:	search12
Date/Time done:	Mon Feb 13 20:05:08 2023
Database(s):	CSD version 5.43 updates (Mar 2022)<br>CSD version 5.43 (November 2021)<br>CSD version 5.43 updates (Jun 2022)<br>CSD version 5.43 updates (Sep 2022)<br>CSD version 5.43 updates (Nov 2022)
Restriction Info:	No refcode restrictions applied
Filters:	None
Percentage Completed:	100%
Number of Hits:	17

Single query used. Search found structures that:

match

Query 2

Query 2

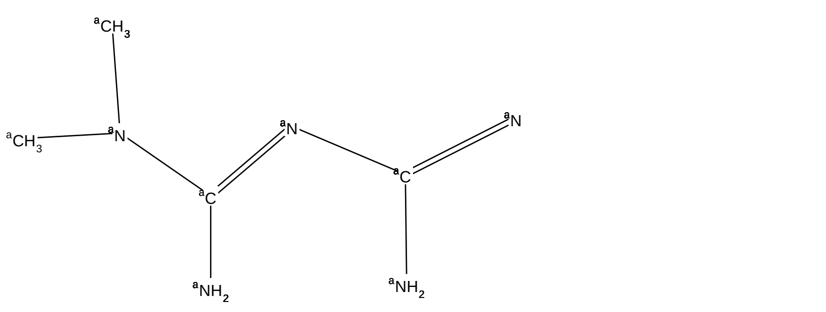

Search: search12 (Mon Feb 13 20:05:08 2023): Hits 1-4

MAXJAA

Reference: Jingjing Sun, Lina Jia, Mengwei Wang, Yu Liu, Min Li, Dandan Han, Junbo Gong (2022) *Cryst.Growth Des.* ,**22,**1005

Formula: $C_{15} H_{12} N_1 O_3 S_2{}^{1-}, C_4 H_{12} N_5{}^{1+}, C_3 H_6 O_1$

Compound Name: amino{[amino(dimethylamino)methylidene]amino}methaniminium [5-(2-methyl-3-phenylprop-2-en-1-ylidene)-4-oxo-2-sulfanylidene-1,3-thiazolidin-3-yl]acetate acetone solvate

Synonym: metformin epalrestat acetone solvate

Space Group:	P-1	***Cell:***	***a*** 7.588(0)	***b*** 9.321(0)	***c*** 19.335(0)
Space Group No.:	2	***(Å,°)***	α 96.17(0)	β 97.92(0)	γ 108.14(0)

R-Factor (%): 3.25 ***Temperature(K):*** 123 ***Density(g/cm^3):*** 1.324

Parameters

Fragment 1
TOR1 (T) -156.076

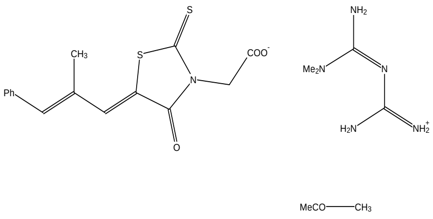

DEXBUF

Reference: J.B.Nanubolu, B.Sridhar, K.Ravikumar, K.D.Sawant, T.A.Naik, L.N.Patkar, S.Cherukuvada, B.Sreedhar (2013) *CrystEngComm* ,**15,**4448

Formula: $C_{23} H_{14} O_6{}^{2-}, 2(C_4 H_{12} N_5{}^{1+})$

Compound Name: bis(Metformin) embonic acid

Space Group:	P-1	***Cell:***	***a*** 7.468(0)	***b*** 13.802(1)	***c*** 16.286(1)
Space Group No.:	2	***(Å,°)***	α 73.33(0)	β 83.17(0)	γ 82.67(0)

R-Factor (%): 3.63 ***Temperature(K):*** 294 ***Density(g/cm^3):*** 1.352

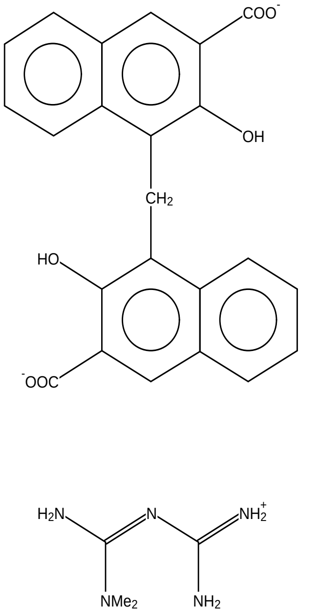

Parameters

Fragment 1
TOR1 (T) 147.696

Fragment 2
TOR1 (T) 170.170

DEXBUF01

Reference: J.B.Nanubolu, B.Sridhar, K.Ravikumar, K.D.Sawant, T.A.Naik, L.N.Patkar, S.Cherukuvada, B.Sreedhar (2013) *CrystEngComm* ,**15,**4448

Formula: $C_{23} H_{14} O_6{}^{2-}, 2(C_4 H_{12} N_5{}^{1+})$

Compound Name: bis(Metformin) embonic acid

Space Group:	P21/c	***Cell:***	***a*** 17.810(0)	***b*** 7.928(0)	***c*** 25.802(0)
Space Group No.:	14	***(Å,°)***	α 90.00	β 119.50(0)	γ 90.00

R-Factor (%): 4.39 ***Temperature(K):*** 294 ***Density(g/cm^3):*** 1.355

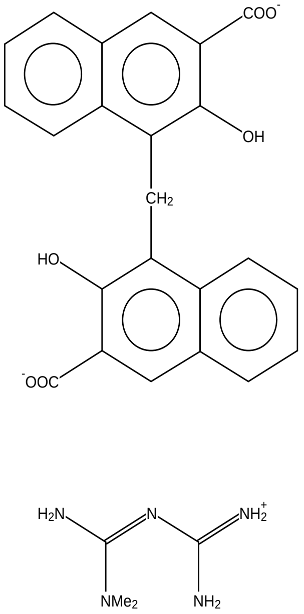

Parameters

Fragment 1
TOR1 (T) -146.972

Fragment 2
TOR1 (T) -153.477

EQUTIV

Reference: R.Olar, M.Badea, M.N.Grecu, C.-M.Balotescu, D.Marinescu, E.-E.Iorgulescu, V.Lazar, C.Bleotu (2010) *Analele Univ. Bucuresti Chim.* ,**19,**13

Formula: $2(C_4 H_{12} N_5{}^{1+}), Cl_1 O_4{}^{1-}, N_1 O_3{}^{1-}$

Compound Name: bis(amino((amino(dimethylamino)methylene)amino)methaniminium) nitrate perchlorate

Space Group:	C2/c	***Cell:***	***a*** 7.147(6)	***b*** 25.460(20)	***c*** 10.951(9)
Space Group No.:	15	***(Å,°)***	α 90.00	β 98.24(1)	γ 90.00

R-Factor (%): 6.82 ***Temperature(K):*** 297 ***Density(g/cm^3):*** 1.421

Parameters

Fragment 1
TOR1 (T) 25.785

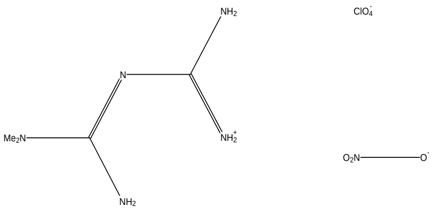

Search: search12 (Mon Feb 13 20:05:08 2023): Hits 5-8

EWISAH

Reference: J.A.Polito-Lucas, J.A.Nunez-Avila, S.Bernes, A.Perez-Benitez (2021) *IUCrData* ,**6,**x210634

Formula: $O_{28}\,V_{10}\,^{6-}$,2($C_4\,H_{12}\,N_5\,^{1+}$),4($H_4\,N_1\,^{1+}$),6($H_2\,O_1$)

Compound Name: tetrakis(ammonium) bis(metforminium) icosakis(μ-oxo)-octaoxo-deca-vanadium hexahydrate

Space Group:	P-1	***Cell:***	***a*** 9.796(0)	***b*** 10.101(0)	***c*** 13.097(0)		
Space Group No.:	2	***(Å,°)***	α 81.08(0)	β 70.91(0)	γ 63.32(0)		

R-Factor (%): 2.54 ***Temperature(K):*** 295 ***Density(g/cm3):*** 2.121

Parameters

Fragment 1
TOR1 (T) -154.277

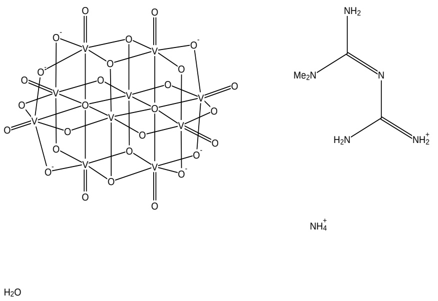

JUMXOH

Reference: Xufei Bian, Lan Jiang, Jing Zhou, Xiaoshu Guan, Jingyu Wang, Peng Xiang, Junyi Pan, Xiangnan Hu (2020) *Molecules* , **25,**1343

Formula: $C_{18}\,H_{18}\,N_3\,O_3\,S_1\,^{1-}$,$C_4\,H_{12}\,N_5\,^{1+}$

Compound Name: amino{[amino(dimethylamino)methylidene]amino}methaniminium 5-[(4-{2-[methyl(pyridin-2-yl)amino]ethoxy}phenyl)methyl]-2,4-dioxo-1,3-thiazolidin-3-ide

Synonym: metformin rosiglitazone

Space Group:	P-1	***Cell:***	***a*** 6.948(0)	***b*** 11.315(0)	***c*** 17.241(0)
Space Group No.:	2	***(Å,°)***	α 72.38(0)	β 82.37(0)	γ 74.15(0)

R-Factor (%): 5.23 ***Temperature(K):*** 293 ***Density(g/cm3):*** 1.302

Parameters

Fragment 1
TOR1 (T) 24.784

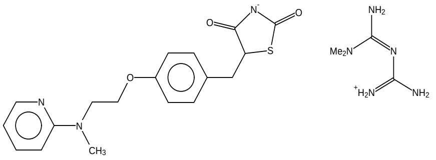

NAKWAB

Reference: N.K.Manjunatha, Mahesha, B.H.Gayathri, N.K.Lokanath, M.T.Swamy, S.Chandra Nayaka, B.P.Siddaraju, N.Ragini, M.AL-Ghorbani, B.R.Kannika, S.Madan Kumar (2020) *Chem. Data Collect.* ,**30,**100577

Formula: $C_4\,H_{12}\,N_5\,^{1+}$,$C_6\,H_2\,N_3\,O_7\,^{1-}$

Compound Name: 2-(amino(iminio)methyl)-1,1-dimethylguanidine 2,4,6-trinitrophenolate

Synonym: metformin picrate

Space Group:	P21/c	***Cell:***	***a*** 11.476(1)	***b*** 5.644(0)	***c*** 24.213(3)
Space Group No.:	14	***(Å,°)***	α 90.00	β 94.29(0)	γ 90.00

R-Factor (%): 5.83 ***Temperature(K):*** 293 ***Density(g/cm3):*** 1.522

Parameters

Fragment 1
TOR1 (T) -18.142

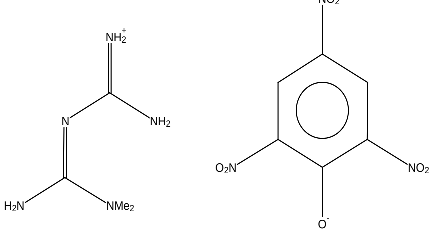

NICCEJ

Reference: J.Satyanarayana Reddy, N.Ravikumar, G.Gaddamanugu, K.N.Naresh, S.S.Rajan, K.Anand Solomon (2013) *J.Mol.Struct.* ,**1039,** 137

Formula: $C_{24}\,H_{25}\,O_6\,^{1-}$,$C_4\,H_{12}\,N_5\,^{1+}$

Compound Name: Amino((amino(dimethylamino)methylene)amino)methaniminium 6,8-dihydroxy-2-methoxy-1,7-bis(3-methylbut-2-en-1-yl)-9-oxo-9H-xanthen-3-olate

Space Group:	P21/n	***Cell:***	***a*** 12.053(5)	***b*** 11.729(5)	***c*** 20.653(5)
Space Group No.:	14	***(Å,°)***	α 90.00	β 101.45(0)	γ 90.00

R-Factor (%): 4.51 ***Temperature(K):*** 293 ***Density(g/cm3):*** 1.253

Parameters

Fragment 1
TOR1 (T) 155.712

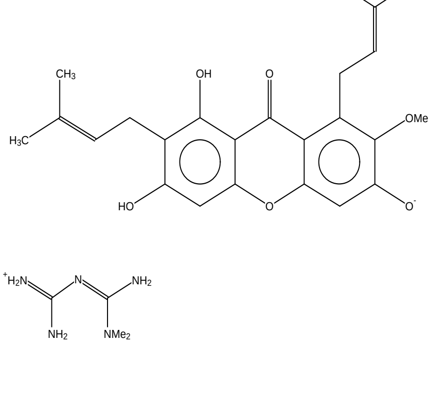

Search: search12 (Mon Feb 13 20:05:08 2023): Hits 9-12

NUPXED

Reference: J.Dong, B.Liu, B.Yang (2015)
Acta Crystallogr.,Sect.E:Cryst.Commun. ,**71,**o747

Formula: $2(C_4 H_{12} N_5{}^{1+}),2(C_1 H_4 O_1),C_1 O_3{}^{2-}$

Compound Name: bis(amino((amino(dimethylamino)methylene)amino)methaniminium) carbonate methanol solvate

Space Group:	C2/c	***Cell:***	***a***	13.573(1)	***b***	10.563(0)	***c***	13.982(1)
Space Group No.:	15	***(Å,°)***	α	90.00	β	90.39(0)	γ	90.00

R-Factor (%): 8.26 ***Temperature(K):*** 298 ***Density(g/cm3):*** 1.274

Parameters

Fragment 1
TOR1 (T) 17.196

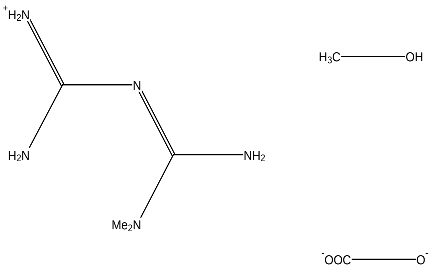

OJOSUC

Reference: R.Olar, M.Badea, D.Marinescu, C.-M.Chifiriuc, C.Bleotu, M.N.Grecu, E.E.Iorgulescu, M.Bucur, V.Lazar, A.Finaru (2010)
Eur.J.Med.Chem. ,**45,**2868

Formula: $C_4 H_{12} N_5{}^{1+},C_2 H_3 O_2{}^{1-}$

Compound Name: N,N-dimethylbiguanidinium acetate

Space Group:	P21/n	***Cell:***	***a***	9.993(1)	***b***	8.894(0)	***c***	10.703(1)
Space Group No.:	14	***(Å,°)***	α	90.00	β	91.14(0)	γ	90.00

R-Factor (%): 6.56 ***Temperature(K):*** 297 ***Density(g/cm3):*** 1.321

Parameters

Fragment 1
TOR1 (T) -160.747

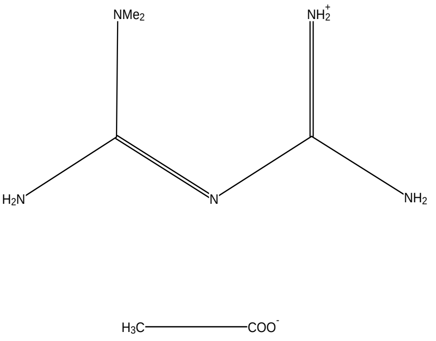

OJOSUC01

Reference: Xiaodan Wei, Yuhua Fan, Caifeng Bi, Xingchen Yan, Xia Zhang, Xin Li (2014) *Bull.Korean Chem.Soc.* ,**35,**3495

Formula: $C_4 H_{12} N_5{}^{1+},C_2 H_3 O_2{}^{1-}$

Compound Name: N,N-dimethylbiguanidinium acetate

Space Group:	P21/n	***Cell:***	***a***	10.001(0)	***b***	8.906(0)	***c***	10.703(0)
Space Group No.:	14	***(Å,°)***	α	90.00	β	91.31(0)	γ	90.00

R-Factor (%): 4.26 ***Temperature(K):*** 298 ***Density(g/cm3):*** 1.319

Parameters

Fragment 1
TOR1 (T) -160.899

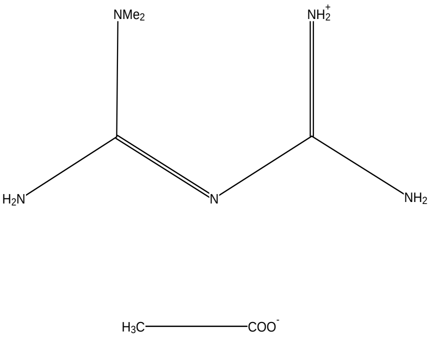

QILBOF

Reference: E.Sanchez-Lara, S.Trevino, B.L.Sanchez-Gaytan, E.Sanchez-Mora, M.E.Castro, F.Melendez-Bustamante, M.A.Mendez-Rojas, E.Gonzalez-Vergara (2018)
Frontiers in Chemistry ,**6,**402

Formula: $O_{28} V_{10}{}^{6-},6(C_4 H_{12} N_5{}^{1+}),6(H_2 O_1)$

Compound Name: hexakis(amino{[amino(dimethylamino)methylidene]amino}methaniminium) icosakis(μ-oxido)-octaoxo-deca-vanadium(v) hexahydrate

Synonym: hexakis(metforminium) icosakis(μ-oxido)-octaoxo-deca-vanadium(v) hexahydrate

Space Group:	P-1	***Cell:***	***a***	11.562(0)	***b***	13.279(0)	***c***	13.769(0)
Space Group No.:	2	***(Å,°)***	α	96.01(0)	β	107.35(0)	γ	115.56(0)

R-Factor (%): 5.09 ***Temperature(K):*** 293 ***Density(g/cm3):*** 1.750

Parameters

Fragment 1
TOR1 (T) -162.291

Fragment 2
TOR1 (T) 150.600

Fragment 3
TOR1 (T) 156.245

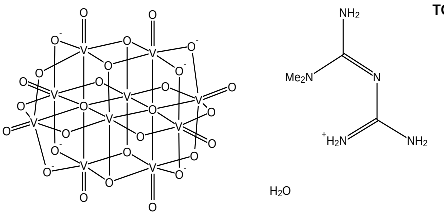

Search: search12 (Mon Feb 13 20:05:08 2023): Hits 13-16

ROLFUW

Reference: L.Jia, S.Wu, J.Gong (2019) *Acta Crystallogr.,Sect.C:Cryst.Struct.Chem.* ,**75,**1250

Formula: $C_{12} H_{17} N_2 O_3 S_1$ $^{1-}$,$C_4 H_{12} N_5$ $^{1+}$

Compound Name: amino{[amino(dimethylamino)methylidene]amino}methaniminium (butylcarbamoyl)(4-methylbenzene-1-sulfonyl)azanide

Synonym: metformin tolbutamide

Space Group:	P21/n	***Cell:***	***a*** 7.985(1)	***b*** 28.569(6)	***c*** 8.826(1)
Space Group No.:	14	***(Å,°)***	α 90.00	β 91.61(3)	γ 90.00

R-Factor (%): 5.79 ***Temperature(K):*** 113 ***Density(g/cm^3):*** 1.318

Parameters

Fragment 1
TOR1 (T) -15.057

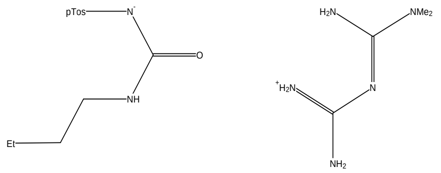

UKODUW

Reference: Wenquan Feng (2020) *CSD Communication(Private Communication)* ,

Formula: $C_{14} H_{10} Cl_2 N_1 O_2$ $^{1-}$,$C_4 H_{12} N_5$ $^{1+}$

Compound Name: amino{[amino(dimethylamino)methylidene]amino}methaniminium [2-(2,6-dichloroanilino)phenyl]acetate

Synonym: diclofenac metformin

Space Group:	P21/c	***Cell:***	***a*** 8.876(0)	***b*** 10.182(1)	***c*** 22.402(2)
Space Group No.:	14	***(Å,°)***	α 90.00	β 94.21(0)	γ 90.00

R-Factor (%): 5.54 ***Temperature(K):*** 296 ***Density(g/cm^3):*** 1.399

Parameters

Fragment 1
TOR1 (T) -158.821

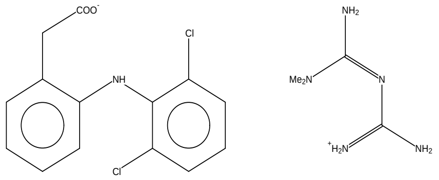

WIBSIJ

Reference: P.Lemoine, A.Tomas, B.Viossat, N.-H.Dung (1994) *Acta Crystallogr.,Sect.C:Cryst.Struct.Commun.* ,**50,**1437

Formula: $C_4 H_{13} N_5$ $^{2+}$,$Cl_4 Cu_1$ $^{2-}$

Compound Name: 1,3-Diamino-1-dimethylamino-3-iminio-2-azoniapropene tetrachloro-copper(ii)

Synonym: N,N-Dimethylimidodicarbonimidic diamide tetrachloro-copper(ii), Metformine tetrachloro-copper(ii)

Space Group:	P21/a	***Cell:***	***a*** 11.128(5)	***b*** 7.926(3)	***c*** 14.735(8)
Space Group No.:	14	***(Å,°)***	α 90.00	β 108.10(5)	γ 90.00

R-Factor (%): 4.60 ***Temperature(K):*** 295 ***Density(g/cm^3):*** 1.810

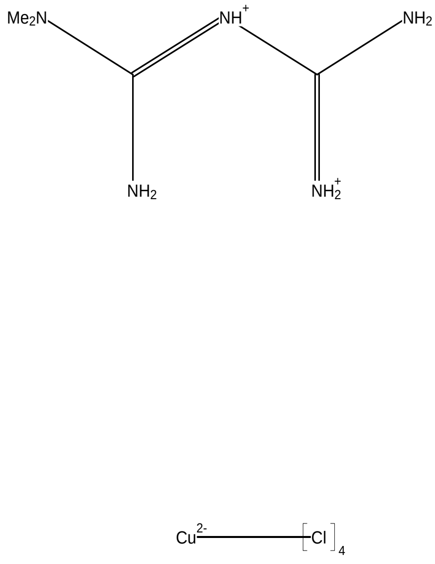

Parameters

Fragment 1
TOR1 (T) -154.307

DELKAK

Reference: L.F.Diniz, P.S.Carvalho, J.E.Goncalves, R.Diniz, C.Fernandes (2022) *New J.Chem.* ,**46,**13725

Formula: $C_4 H_{12} N_5$ $^{1+}$,$C_3 H_3 O_4$ $^{1-}$

Compound Name: amino{[amino(dimethylamino)methylidene]amino}methaniminium carboxyacetate

Synonym: Metformin malonate

Space Group:	P21/c	***Cell:***	***a*** 12.538(0)	***b*** 5.006(0)	***c*** 17.612(0)
Space Group No.:	14	***(Å,°)***	α 90.00	β 91.97(0)	γ 90.00

R-Factor (%): 3.89 ***Temperature(K):*** 293 ***Density(g/cm^3):*** 1.402

Parameters

Fragment 1
TOR1 (T) 33.221

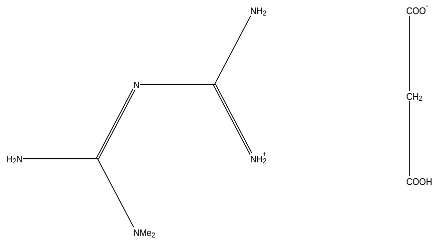

Search: search12 (Mon Feb 13 20:05:08 2023): Hit 17

YEJVOC

Reference:	Lan Jiang, Xiangnan Hu, Linhong Cai (2022) *Molecules* , **27,**3472
Formula:	$C_4 H_{12} N_5{}^{1+}, C_6 H_5 O_5 S_1{}^{1-}$
Compound Name:	amino{[amino(dimethylamino)methylidene]amino}methaniminium 2,5-dihydroxybenzene-1-sulfonate
Synonym:	metformin 2,5-dihydroxybenzenesulfonic acid

Space Group:	C2/c	***Cell:***	***a***	10.876(0)	***b***	10.904(0)	***c***	24.643(0)
Space Group No.:	15	***(Å,°)***	α	90.00	β	93.86(0)	γ	90.00

R-Factor (%): 4.24 ***Temperature(K):*** 293 ***Density(g/cm³):*** 1.455

Parameters

Fragment 1

TOR1 (T) -27.003

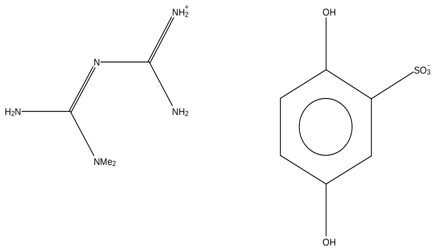

Supplement: Supplementary file 3 [file e-79-00367-sup3.pdf]
